# Enhanced bioelectrochemical degradation of Thiabendazole using biostimulated Tunisian hypersaline sediments: kinetics, efficiency, and microbial community shifts

**DOI:** 10.3389/fmicb.2024.1529841

**Published:** 2025-01-06

**Authors:** Nesrine Saidi, Benjamin Erable, Luc Etchevery, Ameur Cherif, Habib Chouchane

**Affiliations:** ^1^ISBST, BVBGR-LR11ES31, Biotechpole Sidi Thabet, University of Manouba, Ariana, Tunisia; ^2^Laboratoire de Génie Chimique, CNRS, INPT, UPS, Université de Toulouse, Toulouse, France

**Keywords:** bioremediation, bacterial shifts, microbial degradation, microbial community, recalcitrant organic compound, bioelectrodegradation

## Abstract

Thiabendazole (TBZ), a recalcitrant fungicide, is frequently applied in postharvest fruit treatment and generates significant volumes of industrial wastewater (WW) that conventional treatment plants cannot handle. This explores a bioelectrochemical system (BES) for TBZ degradation using Tunisian hypersaline sediments (THSs) as inoculum. Four sets of BES, along with biological controls, were tested using THS subjected to different levels of TBZ biostimulation. Sediments underwent one, two, or three biostimulation phases with increasing TBZ concentrations (0, 10, 100, and 300 mg kg^−1^). Potentiostatic control was applied to BES, polarized at 0.1 V vs. saturated calomel reference electrode (SCE), with a carbon felt working electrode (72 cm^2^ L^−1^) and maintained at 25°C. While current production was very low, sediments biostimulated with 100 mg kg^−1^ kg TBZ produced the highest current density (3.2 mA m^−2^), a 5-fold increase over untreated sediments (0.6 mA m^−2^). GC-FID analysis showed >99% TBZ degradation in all reactors. The TBZ half-elimination time from 27 days with biological treatments to 19 days in BES and further to 6 days following biostimulation. Bacterial analysis revealed a substantial microbial community shift after biostimulation, with a reduction in Bacillota (−64%) and an increase in Proteobacteria (+62%), dominated by *Pseudomonas* (45%) and *Marinobacter* (16%). These findings provide insight into the selective potential of biostimulation cycles to enhance microbial community composition and improve BES performance for TBZ wastewater treatment.

## 1 Introduction

Bioelectrochemical systems (BESs) are emerging as a promising technology for wastewater treatment, offering a dual benefit of environmental cleanup and renewable energy generation. BESs are particularly effective at generating renewable energy, such as direct electricity, methane, and hydrogen, by converting the chemical energy of organic compounds into electrical energy using the catalytic activity of specific electroactive microorganisms (Cruz Viggi et al., 2019; Zheng et al., 2020; Dubuit et al., 2023; Kong et al., 2023). This approach not only supports long-term operation with minimal maintenance costs but also reduces the expenses associated with wastewater (WW) treatment (Wang et al., 2022).

Recently, BESs have demonstrated significant potential for applications beyond conventional WW treatment, particularly in the breakdown of persistent organic pollutants. Bioelectroremediation has become an area of growing interest, effectively addressing various pollutants in wastewater, including phenolic compounds (Ailijiang et al., 2021), antibiotics (Zhang et al., 2024), dyes (Saadaoui et al., 2023b), aromatic hydrocarbons (Hao et al., 2020) and pesticides (Saidi et al., 2022). The rising dependency on water in industrial processes has exacerbated water scarcity due to intense anthropogenic activity (Mancosu et al., 2015). In this context, BESs offer a promising strategy for not only recovering water resources but also converting toxic compounds into safe by-products, paving the way for sustainable environmental remediation.

However, untreated WW from human activities, which is often discharged into rivers and seas, remains a significant source of water quality degradation (Naik and Jujjavarapu, 2021). Among the various pollutants, pesticide residues represent a critical environmental challenge due to their widespread presence and toxicity (European Commission, 2019). Thiabendazole (TBZ) is a benzimidazole fungicide that is frequently detected in fruits packaging WW (FPWW) as it is commonly used during postharvest treatment to eradicate fungi infestations. Up to 2,000 ppm of TBZ solutions are used in the dipping procedure, leading to residual TBZ levels in fruits that can reach up to 3.2 mg kg^−1^ (Portilla-Sangabriel et al., 2022). TBZ is transferred into the water during washing, creating wastewater with high TBZ concentrations. TBZ has been detected in surface water worldwide, with concentrations ranging from 0.9 to 330 ng L^−1^ across regions including Africa, China, Europe, and the United States (Pérez et al., 2013; Odendaal et al., 2015; Fang et al., 2019). Therefore, several methods have been developed to clean these effluents. Advanced oxidation processes (AOPs) are the fastest-growing technique for treating industrial WW with a significant challenge for industrial-scale application due to their high operating cost (Babu Ponnusami et al., 2023). As examples of AOPs processes, photocatalytic nanofiltration reactor (Theodorakopoulos et al., 2023), photocatalytic process, operated in heterogeneous systems (Ranjbari et al., 2022) integrated into two different processes to enhance the treatment efficiency. However, many challenges occur once they are implemented. Currently, some treatment technologies are at an operating industrial scale, including the As-Samra WW Treatment Plant in Jordan (Al-Mashaqbeh et al., 2019) which examined the occurrence and removal efficiency of pharmaceuticals and personal care products using a combined membrane bioreactor reverse osmosis**/**nanofiltration systems; however, the authors reported a negative removal efficiency rate for TBZ. Another study demonstrating the efficient removal of 99% of TBZ using a solar photo-Fenton treatment (Carra et al., 2015) in a raceway pond reactor in an agro-food industry enriched with 100 μg L^−1^ of the fungicide, although they did not mention the possibility of generating hydroxyl radicals, the amount of energy consumed, and the operating expense required for this treatment. Biological processes for TBZ degradation have also been explored. For example, Perruchon et al. (2017) isolated a bacterial consortium able to degrade TBZ, and bioaugmentation approaches have been applied to enhance this process (Papadopoulou et al., 2018). In addition, combined treatment processes are shown to be a promising alternative to treat WW. Combining biological processes with AOPs has shown promise for achieving sustainable degradation of toxic pollutants while reducing costs and chemical consumption (Nidheesh et al., 2022).

Previously, we demonstrated combining electrochemical techniques with biological processes for treating synthetic FPWW (SFPWW) containing TBZ (Saidi et al., 2022). The study highlighted the potential of BESs inoculated with halotolerant bacteria under weak electrostimulation for TBZ remediation in an aqueous solution. This research underscored the utility of Tunisian hypersaline sediments (THSs) for developing bioanodes tailored for persistent pollutant biodegradation. Despite these promising results, further studies are needed to enhance the efficiency of TBZ degradation and detoxification.

We focused on optimizing the bioelectroremediation process to address this knowledge gap by employing biostimulation. This is intended to boost the growth and metabolism of microorganisms in regard to TBZ. Biostimulation is a method that involves enhancing the activity of indigenous microorganisms to increase their ability to break down pollutants in a contaminated area for remediation purposes. Several studies have demonstrated the positive impact of using biostimulation for the bioremediation of many contaminants, such as diesel compounds (Meyer et al., 2018), chlorinated hydrocarbon (Dutta et al., 2022) and pesticides (Raffa and Chiampo, 2021). We hypothesize that biostimulation can improve BES performance for TBZ degradation by enhancing microbial activity and community structure.

This study aimed to evaluate the impact of biostimulation on the THS microbial consortium for enhancing TBZ degradation in BESs and biological controls (BIOs) using SFPWW. Taxonomic analysis was performed to assess microbial community shifts following TBZ exposure, and analytical techniques were used to evaluate TBZ degradation and detoxification. This research provides insights into optimizing BESs for treating TBZ-contaminated wastewater, advancing the field of bioelectroremediation.

## 2 Materials and methods

### 2.1 Chemicals

An analytical standard of TBZ (≥99% purity, Sigma–Aldrich) was used for analytical purposes, and a suitable bioreagent of TBZ (T5535, Sigma–Aldrich) was also used for the experiments. Dichloromethane (DCM stabilized with ~20 ppm for amylene) for TBZ extraction was purchased from AppliChem (75–09-2).

### 2.2 Sample collection and physicochemical analysis

THS used as a source of halophilic microorganisms were collected from a hypersaline lake, Chott El-Djerid (CJ) located in the south of Tunisia (N: 33° 57.272′ E: 008° 24.393′) in September 2023. Samples were mixed, secured in sterile bags, and stored at +4°C until experiments were run. The physicochemical analyses of conductivity, pH, total organic carbon, and PO_4_^3−^, SO_4_^2−^, Na^+^, K^+^, Ca^2+^, Mg^2+^, Fe^2+^, and Cl^−^ the content of the sediments sample were carried out using conventional methods, in a specialized environmental analysis laboratory, Mediterranean Center for Environmental and Industrial Analysis (CMA AZUR LAB).

### 2.3 Biostimulation strategy of Chott El-Djerid sediment (CJS)

A bulk of sediment samples were used without biostimulation, which has no prior hint of TBZ exposure serving as a reference point for comparison (CJS-0). Then, a series of three stimulations were conducted, each involving the addition of different concentrations of a TBZ, spaced 15 days apart between treatments, as indicated below. TBZ solutions were prepared by diluting the stock solution in deionized water according to the following formula:


$$\left[\text{TBZi}\;\left(\text{ppm}\right)\right]=\frac{\left[\;TBZ\;\left(\text{mg}\;{\text{kg}}^{-1}\right)]\times m_{sed}(\text{kg}\right)}{V\left(L\right)}$$

where TBZi is the initial concentration of TBZ solution, TBZ is the concentration of thiabendazole in sediment (mg kg^−1^), *m*_sed_ is the mass of sediment that has been treated in kg, and *V* is the volume (in L) of the TBZ-prepared solution.

**Figure 1:**
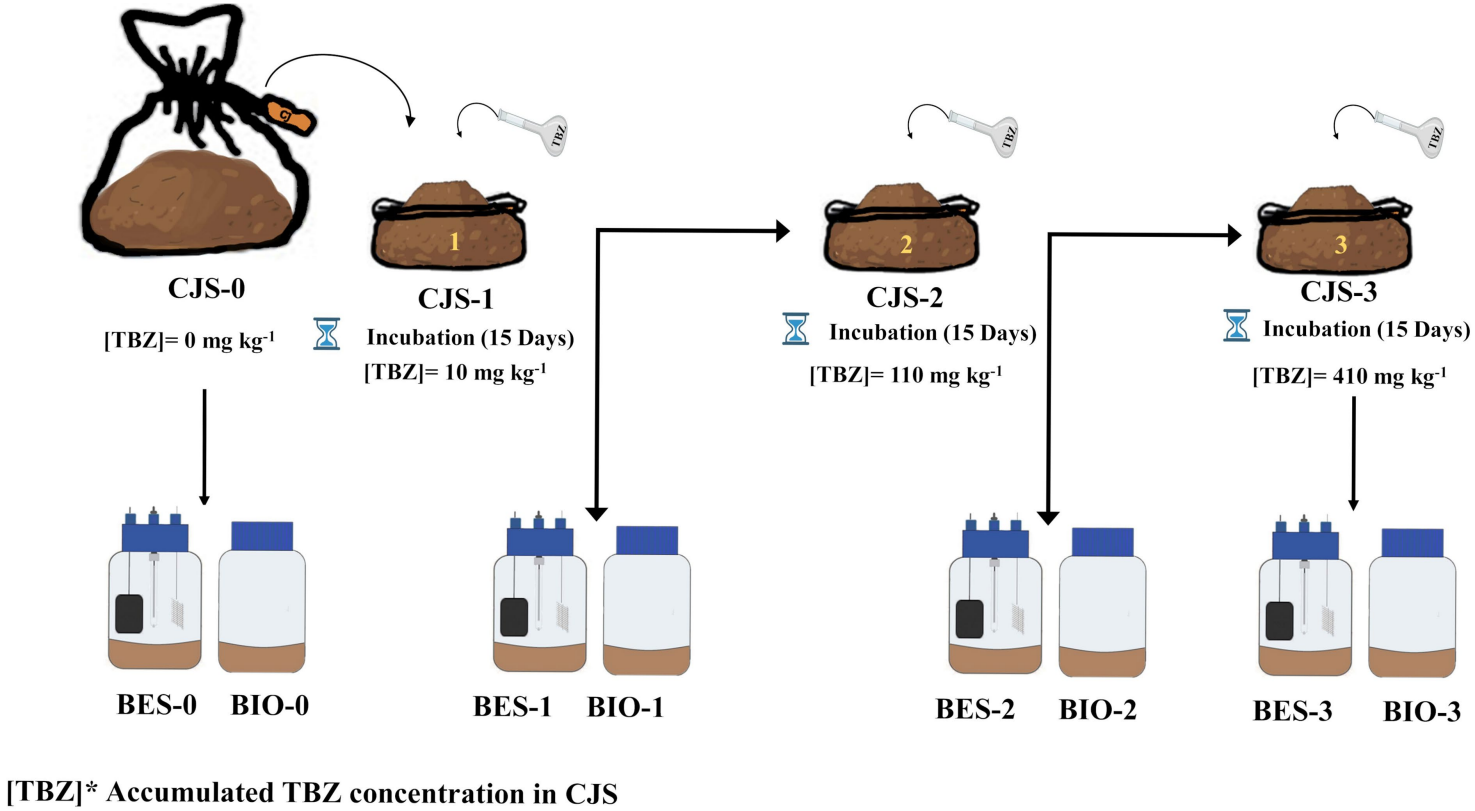
Schematic illustration for biostimulation strategy of Chott El-Djerid sediment (CJS).

The first biostimulation step was to stimulate the microbial community within sediments by introducing an initial concentration of 10 mg kg^−1^ for CJS-1. In the second phase of biostimulation, 100 mg kg^−1^ was added for CJS-2. Finally, in the third stimulation phase, TBZ concentration was increased by incorporating 300 mg kg^−1^ for CJS-3. Figure 1 shows the various biostimulation phases.

### 2.4 Bioelectrochemical experiments

The combined effects of the biological and the electrochemical stimulation of CJS were studied in single-compartment BES (Duran Schott glass 550 mL) set up with a three-electrode system. The working electrode (WE) was made of carbon felt with a geometric area of 36 cm^2^ (6 cm × 6 cm), the platinum counter electrode (CE) was positioned opposite each other, and the saturated calomel reference electrode (SCE) was placed as near as possible to the WE. The WE was polarized at +0.1 V vs. SCE using a multichannel potentiostat (Bio-Logic SA) operated by the EC lab software, and the current was recorded every 20 min.

All the experiments were systematically carried out in triplicate. The reactors were maintained at 25 ± 1°C, the pH was 7 ± 0.1. Each reactor had 20% of CJS either non-biostimulated or biostimulated at different levels, along with 80% of SFPWW. SFPWW is a modified synthetic medium which contains the following substances in g L^−1^: 0.1 KCl, 0.6 NaH_2_PO_4_, 1.5 NH_4_Cl, 2.5 NaHCO_3_, 0.1 MgCl_2_, 0.01 CaCl_2_, and a trace mineral mix (10 mL L^−1^, ATCC MD-TMS) (Bridier et al., 2015). Approximately 30 ppm of glucose (Glu) was added as a co-substrate and 150 ppm of TBZ as the substrate.

Biocontrol experiments (without electrodes) were carried out in parallel with bioelectrochemical experiments under the same conditions. Table 1 summarizes the various BESs, and BIOs carried out in this study. A total of 24 reactors were used to study eight different conditions: BES inoculated with CJS at stimulation levels of 10, 100, and 300 mg kg^−1^ in reactors named BES-0, BES-1, BES-2, and BES-3 with electrodes and BIO-0, BIO-1, BIO-2, and BIO-3 without electrodes, respectively.

**Table 1:** Bioelectrochemical and biological experiments conducted in this study.

Biostimulation step	[TBZ] added to THS (mg kg^−1^)	Reactors code	
		BES	BIO
0	0	BES-0	BIO-0
1	10	BES-1	BIO-1
2	100	BES-2	BIO-2
3	300	BES-3	BIO-3

### 2.5 Anodic biofilm pretreatment and architecture study

After bioelectrochemical TBZ degradation experiments in different BES, chronoamperometry was stopped, and relative bioanodes were investigated using scanning electron microscopy (SEM) to visualize the surface structure and Confocal laser scanning microscopy (CLSM) to analyze the total cells and the exopolymeric matrix of the formed biofilm. For the SEM visualization, samples were fixed with 4% glutaraldehyde, washed, and dehydrated with different concentrations of acetone then left to dry. Prior to the examination, they were metalized with gold (Au) and then observed with Leo 435 VP-Carl Zeiss SMT. For the CLSM analysis, samples were stained with a combination of three fluorescent dyes following the protocol described by Martinez Ostormujof et al. (2023): Concavalin A tetramethylrhodamine conjugate (ConA-TMR, Thermofischer Scientific) at 0.1 g L^−1^ for *α*-polysaccharides, fluorescein isothiocyanate isomer I (FITC, Merck) at 0.05 g L^−1^ for proteins and 4′,6-diamidino-2-phenylindole dihydrochloride (DAPI, Merck) at 1.05 × 10^−4^ g L^−1^ for total cells. After completing 30 min spent in contact with the mixed solution, the carbon-felt electrodes were delicately rinsed with a physiological solution and then observed after 48 h. A Leica SP8-2017 microscope with 10× 0.30^−1^ objective (HC PC FLUOTAR) in dry immersion was used to visualize the samples. Images were acquired by LAS-X software (Leica Microsystems), two stacks of horizontal plane images (1,024 × 1,024 pixels) were captured for each sample, and the *z*-stacks were then registered to build three-dimensional (3D) images of the biofilm using ImageJ’s 3D script plugin (Schindelin et al., 2012).

### 2.6 Glucose and TBZ degradation analysis

#### 2.6.1 TBZ UV–visible analysis

To evaluate the TBZ degradation rate, a calibration curve was established with standard solutions at concentrations of 0, 2, 5, 10, 15, and 20 ppm using Jenway 7315 ultraviolet–visible (UV–Vis) spectrophotometer in the range of 190–350 nm. The characteristic peak of TBZ observed at 298 nm, was used to determine the fungicide’s residual content in various reactors.

#### 2.6.2 Gas chromatography analysis

A gas chromatography (GC) system equipped with a flame ionization detector (FID) was used to consolidate UV–visible results. TBZ was extracted from liquid solution as follows: 3-ml aliquot of each sample was placed in a 10-ml glass centrifuge tube to which 3 mL of DCM was added. The mixture was stirred in a vortex mixer for 1 min, then 2 g of MgSO_4_ and 0.5 g of NaCl were added. After vortexing for 5 min, the extract was centrifuged at 4,000 rpm for 10 min to remove the top layer. Approximately 2 mL of the organic layer extract was transferred into a clean-up tube containing 0.5 g of MgSO_4_, vortexed then centrifuged at 4,000 rpm for 1 min. Finally, 300 μL of the extract was purified through a 0.22 μm filter membrane and analyzed using a Thermo Scientific Trace 1310 GC-FID. Capillary column type CB-CP WAX 52 (25 m × 0.25 mm; film thickness 0.2 μm) using helium as the carrier gas. The GC-FID operating conditions were modified as discussed by Kai et al. (2023) as follows: injector temperature, 280°C; detector temperature, 300°C; oven temperature program—initial temperature 80°C held for 0 min and ramp temperature 10°C min^−1^ to 275°C held for 14 min. The method validation was carried out by linearity, which was evaluated by the correlation coefficient (R^2^) of the matrix-matched calibration curves of the TBZ standard. The accuracy and precision were conducted by recovery experiments at 2, 5, 30, 80, and 120 ppm with triplicates. Based on the results, good linearity was obtained with a determination coefficient value of 0.999.

#### 2.6.3 FTIR spectrometry analysis

TBZ solutions were analyzed before and after the BES degradation study as a way to detect any modifications in the functional groups of the fungicide. Fourier transform infrared spectroscopy (FT-IR) spectra were carried out in the mid-IR region of 450–4,000 cm^−1^ using a PerkinElmer (FTIR 1720-X) spectrometer with 46 scans and 5 cm^−1^ resolution.

#### 2.6.4 COD and glucose measurement

To measure chemical oxygen demand (COD), samples were obtained from SFPWW before and after experiments, treated with chloride elimination kit LCW925 (Hach Lange) to remove chloride ions, and the COD was measured using an LCK 514 kit (Hach Lange, range 100–2,000 mg O_2_ L^−1^) after a 0.5 dilution or an LCI 500 kit (Hach Lange, range 0–150 mg O_2_ L^−1^). The COD removal rate (RR_COD_) was calculated as shown in the following formula:


$${RR}_{\text{COD}}=1-\frac{\text{COD}\;\text{effluent}}{\text{COD}\;\text{influent}}\;\times 100$$

A high-performance liquid chromatography (HPLC) (Thermo Scientific Vanquish, France) instrument equipped with a refractive index detector and an HPX-87H AMINEX column was used for glucose concentration analysis. The mobile phase consisted of 10 mmol of sulfuric acid solution, at a flow rate of 0.6 mL min^−1^ and maintained at 40°C, with an injection volume of 20 μL (Mouguech et al., 2024).

### 2.7 Bacterial diversity analysis

Total DNA was extracted from different non-biostimulated and biostimulated sediments, anolytes, and BIO reactors using PureLink Microbiome DNA Purification Kit (Invitrogen), and from bioanodes with a DNeasy Power Biofilm Kit-QIAGEN. The DNA samples’ qualities and concentrations were checked with a microvolume spectrophotometer (FastGene NanoView Photometer) with an absorbance ratio $A_{\frac{260\;\text{nm}}{280\;\text{nm}}}$. Sequencing of the 16S ribosomal RNA (rRNA) gene of the V4–V5 variable region was performed by MR DNA^1^ (Shallowater, TX, United States) on a MiSeq platform following the manufacturer’s guidelines. Data sequences were processed using the MR DNA analysis pipeline. The operational taxonomic unit (out) taxonomic classification was achieved by comparing the sequences using the Basic Local Alignment Search Tool for nucleotide sequences (BLASTn) against a curated database derived from the National Center for Biotechnology Information (NCBI).^2^

### 2.8 Statistical analysis

All experiments were performed in triplicates. The results are presented as mean ± standard deviation (mean ± SD). To determine the statistical significance of the observed differences between experimental groups, statistical analysis was performed with one-way analysis of variance (ANOVA), with a significance threshold set at *p* < 0.05.

## 3 Results

### 3.1 Physicochemical properties of CJS

The chemical composition and physical characteristics of CJS are given in Table 2. Sediment analysis revealed a conductivity value of 74.1 mS cm^−1^ which is considered high compared to the average seawater conductivity and much higher than that of SFPWW. This elevated conductivity reflects the inoculated sediment’s high salinity, which favors ion flow in the reactor, promoting the extracellular electron transfer from the electroactive biofilm, thus enhancing BES performance. Sediments pH was 7.8 and contained an assortment of ions mainly composed of Ca^2+^, Mg^2+^, and K^+^ with concentrations of 97.8, 32.4, and 71.6 (g kg^−1^ dry matter (DM)), respectively, that are essential for various cellular function such as promoting the growth or metabolic activities of microorganisms as well as their respiratory activities which can work as electron acceptors.

**Table 2:** Basic physicochemical properties of CJ sediments.

Content	Chott El-Djerid sediment (CJS)
pH	7.8
Conductivity (ms cm^−1^)	74.1
Total organic carbon (g kg^−1^ DM*)	19.9
Orthophosphate (mg kg^−1^ DM)	10.19
Chloride (g kg^−1^ DM)	130
Sulfate (g kg^−1^ DM)	9.29
Calcium (g kg^−1^ DM)	97.8
Magnesium (g kg^−1^ DM)	32.4
Potassium (g kg^−1^ DM)	71.6
Sodium (g kg^−1^ DM)	12.9
Iron (g kg^−1^ DM)	18.6

DM*, dry matter.

### 3.2 Electrochemical activity of bioanodes

**Figure 2:**
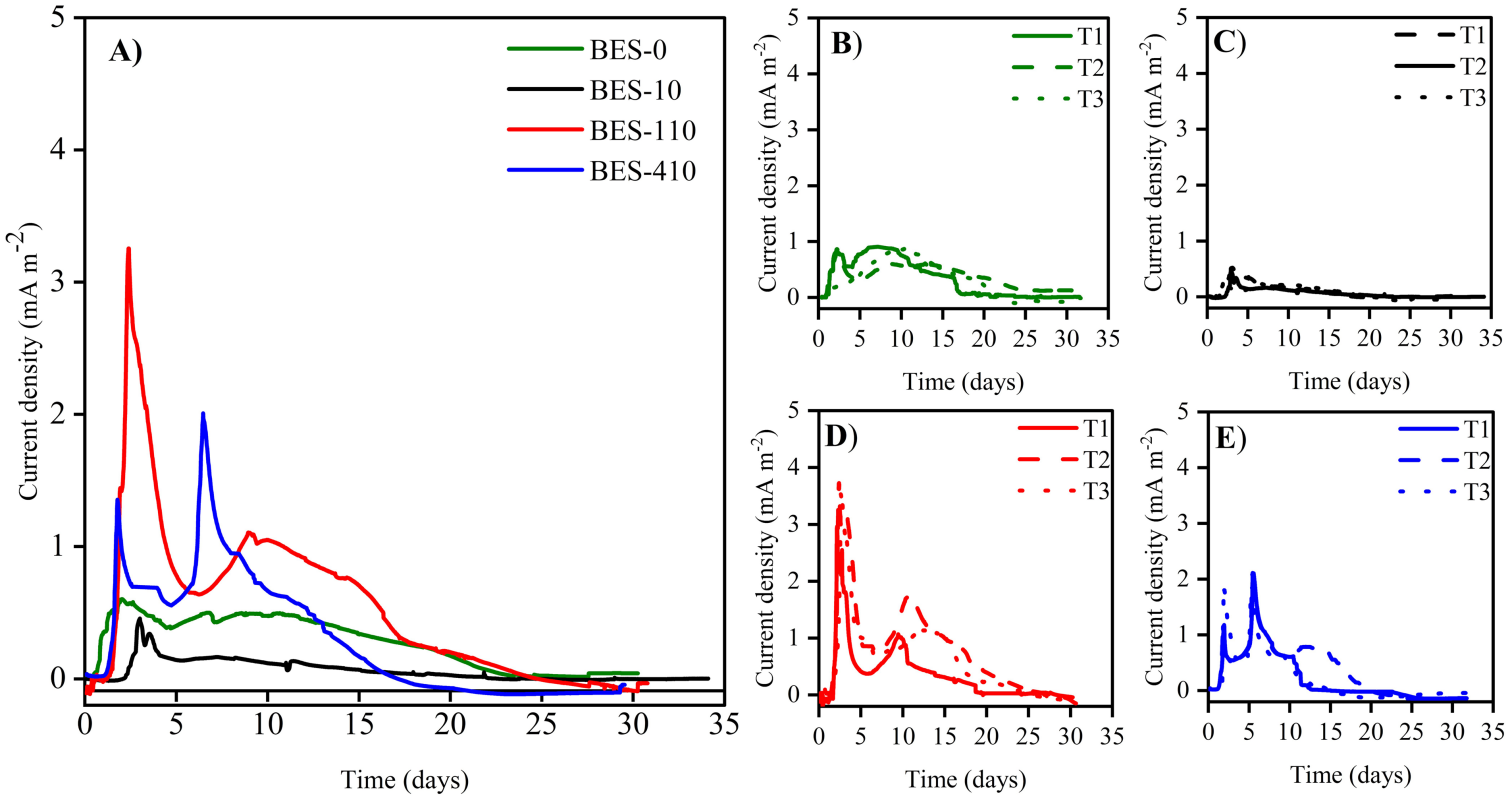
Current densities evolution vs. time of **(A)** different bioelectrochemical systems colonized with Chott El-Djerid sediment (CJS), **(B)** BES-0 inoculated with the non-biostimulated CJS-0, **(C)** BES-1 inoculated with CJS-1, **(D)** BES-2 inoculated with CJS-2, and **(E)** BES-3 inoculated with CJS-3, under the constant potential of 0.1 V vs. SCE, fed with 150 and 30 ppm of TBZ and glucose, respectively.

The non-biostimulated CJS-0 and the biostimulated CJS at different levels of TBZ; CJS-1, CJS-2, and CJS-3, were tested to generate bioanodes in SFPWW. As indicated above, the four sediments were used as inocula to allow biofilm growth on the surface of carbon felt WE. The three replicates of each BES studied simultaneously were under constant polarization of 0.1 V vs. SCE. The current densities recorded for 35 days are displayed in Figure 2. Figure 2A shows the average current density of each BES. Replicates of each experience demonstrate a uniform alignment of the evolution of current densities, indicating consistent experimental conditions (Figures 2B–E). However, slight variations in the current value produced were observed between trials. The maximum currents obtained for BES-0, BES-1, BES-2, and BES-3 were 0.60, 0.39, 3.22, and 2.00 mA m^−2^, respectively, with standard deviation reflecting some variability in current output (±0.11, ±0.07, ±0.58, and ± 0.17). Maximal current densities, cumulated charges, coulombic efficiencies, and degradation half-life of TBZ, COD, and TBZ removals determined from different experiments are summarized in Table 3. After a few days of WE polarization, all reactors’ oxidation currents began to increase in all reactors, and the maximum current densities between 0.39 and 3.22 mA m^−2^ were observed in the first 10 days for all BESs. BES-0 has shown an increase in current reaching a sharp peak after 3 days to attend 0.6 mA m^−2^ corresponding to maximum current density (Figure 2B), followed by a progressive decrease in current production to give a stable value approximately 0.45 mA m^−2^ and a fall to 0.03 mA m^−2^ after 25 days. With the first biostimulation, the chronoamperometry curve from BES-1 demonstrates very low current densities. A detailed evaluation of the BES-2 chronoamperometry (Figure 2D) revealed variations in current density. During the first 5 days, the system produced electricity, reaching a maximum current production with a peak of 3.22 mA m^−2^, which then stabilized, showing a wave with a maximum current of 1.10 ± 0.25 mA m^−2^ on the ninth day. Following the 20th day, there was a considerable drop in current production, which could be explained by the lower availability of electron donors since glucose had been wholly degraded and TBZ concentration in BES-2 was above 32 ppm. On the third biostimulation, BES-3 (Figure 2E) yielded two peaks of current of different intensities, the second peak reaching 2.00 ± 0.17 mA m^−2^, resulting in a 3-fold increase over BES-0.

**Table 3:** Electrochemical and degradation performance of Chott El-Dejrid sediments (CJSs) under different conditions.

	Experiment	TBZ initial concentration (ppm)	TBZ final concentration (ppm)	Removal efficiency of TBZ (%)	Maximum current density (mA m^−2^)	Cumulated charge (coulomb)	$T_{\frac{1}{2}}$ (days)	Initial COD (mg L^−1^)	Final COD (mg L^−1^)	COD_rr_ (%)	Ce (%)
BES	BES-0	150	8.56	94.29	0.60 ± 0.11	3.03	18.60	1959	15	99.23	0.016
	BES-1	150.20	1.22	99.20	0.39 ± 0.07	0.88	9	1959	2	99.90	0.005
	BES-2	153.20	0.35	99.77	3.22 ± 0.58	5.69	5	1964	1	99.95	0.03
	BES-3	156.60	0.25	99.84	2 ± 0.17	3.40	5.80	1970	0	100	0.018
BIO	BIO-0	150	23.91	84.06	–	–	27	1959	41	97.91	–
	BIO-1	150.20	6.22	95.86	–	–	10.80	1959	11	99.44	–
	BIO-2	153.20	2.83	98.15	–	–	5.80	1964	5	99.75	–
	BIO-3	156.60	0.24	97.82	–	–	6.80	1970	0	100	–

### 3.3 Morphological analysis of anodic biofilms

**Figure 3:**
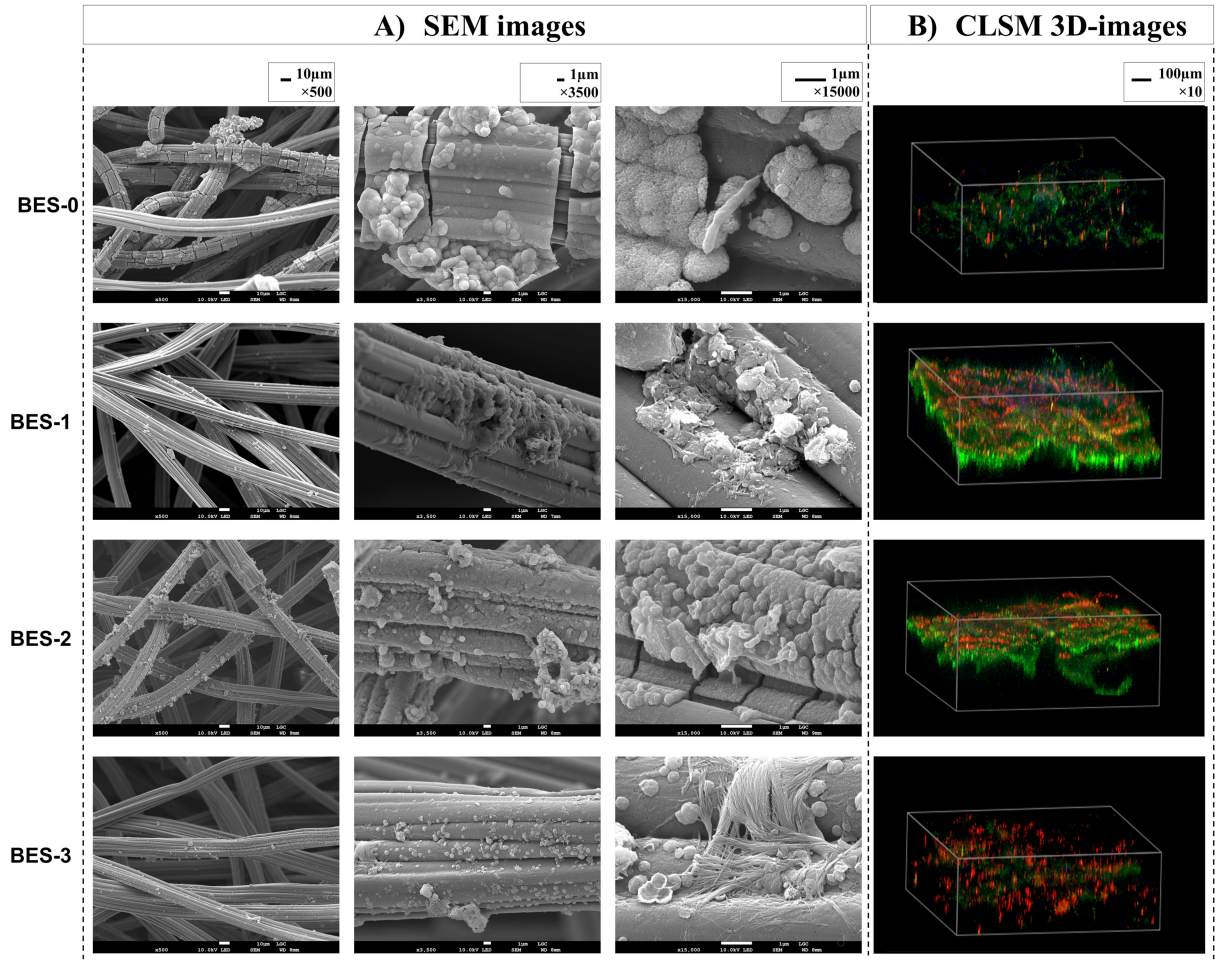
SEM images **(A)** and CLSM 3D images **(B)** of carbon felt bioanodes formed in the different BES.

SEM micrographs and CLSM images of the carbon felt bioanodes recovered from different BESs were analyzed at the end of experiments (Figure 3). Both microscopic images confirmed biofilm development on carbon-felt electrodes. The SEM images showed a few micrometers of thickness biofilms with two different morphologies, the oval-shaped cells—which dominate—and the rod-shaped cells. A closer look (magnification, 15,000×) revealed that the membrane structure of the connected cells on the carbon-felt electrode was surrounded by the biofilm’s extracellular matrix. To characterize the detailed structure of anode surface, CLSM images were captured. 3D images of bioanodes from the four BESs (Figure 3B) illustrated biofilm structure; bacteria labeled with DAPI display blue fluorescence with varying extracellular components, rich in proteins (green fluorescence) and polysaccharides (red fluorescence). BES-0 bioanode structure (Figure 3B) appears the exact amounts of proteins and polysaccharides with a lower density of biofilm. Regarding BES-1 and BES-2 (Figure 3B), bioanodes demonstrated a dense and regular biofilm constructed with two layers, the first corresponding to polysaccharides located close to the adherent bacteria and the second layer with a larger volume of proteins. However, after the final biostimulation stage, that is, BES-3 (Figure 3), a noticeable change in the structure of the biofilm was observed, which now consisted mainly of polysaccharides, indicating a shift toward a more carbohydrate-rich composition.

### 3.4 Glucose and TBZ degradation rate

The initial glucose concentration (30 ppm) added to each BES and biological reactor was completely degraded after 4 days of carbon-felt WE polarization. HPLC analysis (data not shown) revealed a trace of glucose (<0.5 ppm) on days 2 and 3, and no trace from day 4 onward.

**Figure 4:**
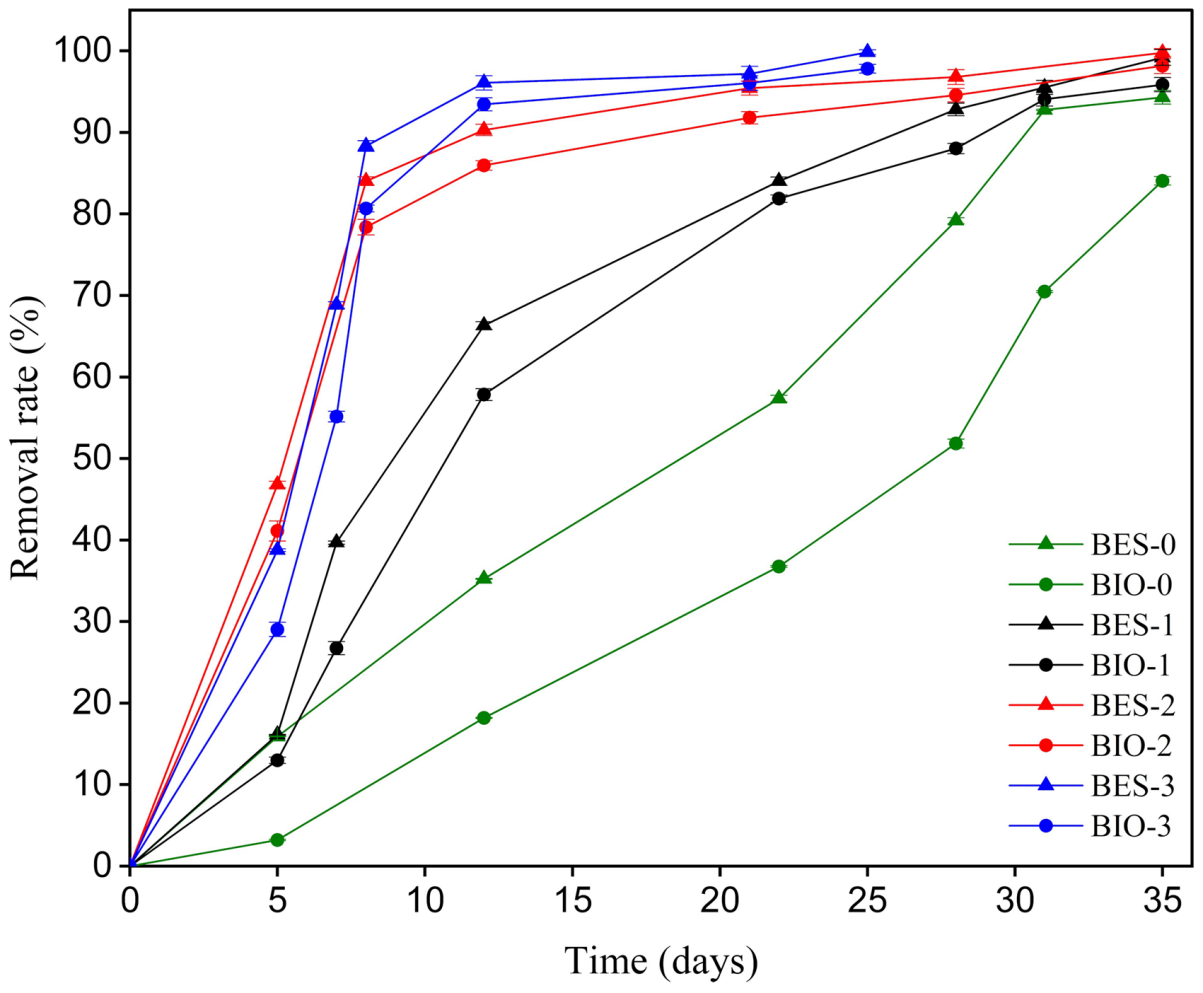
TBZ removal rate profiles, determined by GC-FID analysis in all BES and BIO reactors.

To evaluate the impact of biostimulation and BES treatment on TBZ degradation, the dynamics of TBZ concentration changes were assessed. Figure 4 displays the TBZ removal rate over time in each of the BES and BIO reactors. In addition to the concentration added to the reactors (150 ppm of TBZ), a remaining quantity of TBZ used in CJS pretreatment was present in the reactors. As a consequence, the initial concentration in each reactor was 150 ppm in BES-0 and BIO-0, 150.20 ppm in BES-1 and BIO-1, 153.20 ppm in BES-2 and BIO-2, and 156.60 ppm in BES-3 and BIO-3 (Table 2). Gas chromatography was utilized to verify the UV–Vis data, which revealed the same degradation rate and confirmed the elimination of TBZ in all reactors. From day 5, TBZ concentration was surveyed, showing degradation rates of 3, 16, 13, 16, 41, 47, 29, and 39% in BIO-0, BES-0, BIO-1, BES-1, BIO-2, BES-2, BIO-3, and BES-3, respectively, (Figure 4). BES reactors consistently exhibited greater degradation efficiency compared to BIO reactors. In BES-0, we achieved a TBZ degradation rate approximately 5 times greater than in the BIO-0 compared to BIO-0 after day 5 without using biostimulation pretreatment for CJS. After biostimulation of the CJS, BES, and BIO reactors showed an increased TBZ degradation efficiency in a shorter time. This could be explained by the emergence of a microbial community tolerant to degrade TBZ more efficiently. BES and BIO reactors inoculated with biostimulated sediments had degraded by above 50% within the first 12 days of experiments, proving the effectiveness of biostimulation in enhancing TBZ removal following the third series of biostimulation. Nevertheless, we noticed a significant reduction in TBZ removal rate after 22 days in the reactors inoculated with biostimulated CJS. TBZ concentrations were much lower (less than 20%) at this stage, resulting in slower transport processes. Consequently, its availability in the reactors could become a limiting factor.

### 3.5 TBZ structure modification in BES

**Figure 5:**
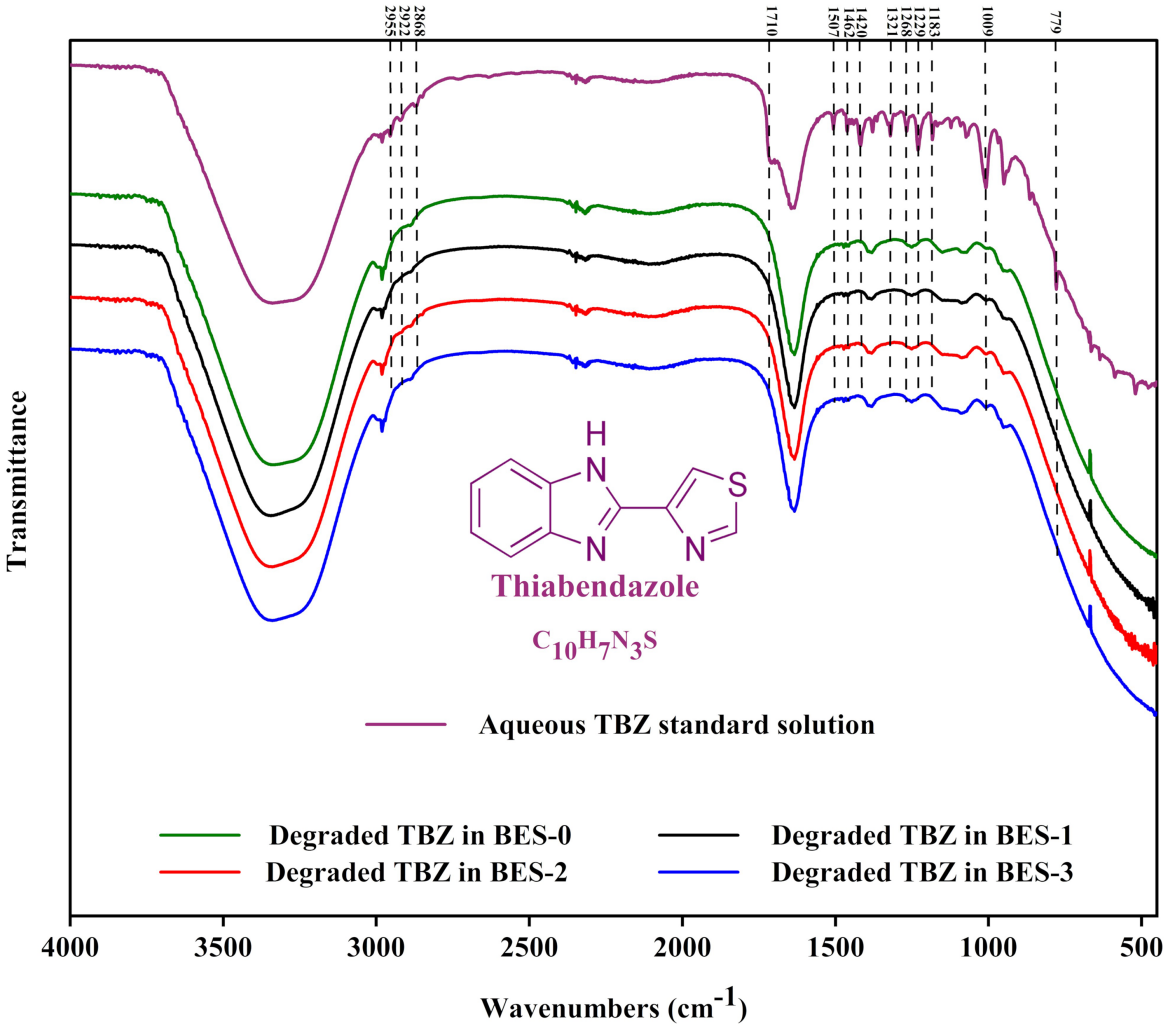
Fourier transform infrared (FTIR) spectrum of undegraded TBZ and degraded TBZ in the bioelectrochemical experiments using different CJS inocula.

FTIR was used to investigate the functional groups of the degraded TBZ after the BES experiment all along with the undegraded fungicide (Figure 5). The FTIR examination of TBZ revealed distinct peaks for aromatic rings (C=C), aliphatic group (-CH_3_), and functional groups (-NH, -CS, and C=N) illustrated from 2,900 to 450 cm^−1^. The band observed at 779 cm^−1^ corresponding to the stretching vibration of the thioether group (-S-) attached to the thiazole ring of TBZ had disappeared after the BES treatment experiment in all reactors. FTIR spectrum also indicates a decrease in peak intensities of the two bands observed at 950 and 1,009 cm^−1^ assigned to C-C stretching vibrations and CH bending vibrations in the benzene ring, respectively. This finding supports the cleavage of TBZ bonds during the experiments. The region from 1,050 to 1,540 cm^−1^ represented in the undegraded TBZ spectra results from C-N stretching of the imidazole ring, benzene ring breathing, and weaker contribution from the asymmetric C-C stretching vibrations of the imidazole-thiazole bond and the benzene ring appeared decreased in the degraded TBZ spectra related to TBZ transformation in the reactors. In addition, peaks spanned in the range of 1,600 and 1740 cm^−1^ that define the interaction vibrations in the imidazole ring containing two nitrogen atoms were not the same before and after TBZ degradation experiments. Moreover, the peak at 1640 cm^−1^ became broader after BES experiments. The generation of intermediate metabolites could explain this. Furthermore, serval bands detected in the region between 2,850 and 2,990 cm^−1^ characterize tertiary amines that disappeared following BES experiments and gave the appearance of a single peak at 2,981 cm^−1^, confirming the breakdown of the molecule. Overall, FTIR spectra provide significant variations in the functional groups following TBZ degradation experiments and support chemical changes in the molecule either by the formation or by the disappearance of specific chemical bonds.

### 3.6 Comparison of the bacterial communities from CJS, bioanodes, anolytes, and BIO

**Figure 6:**
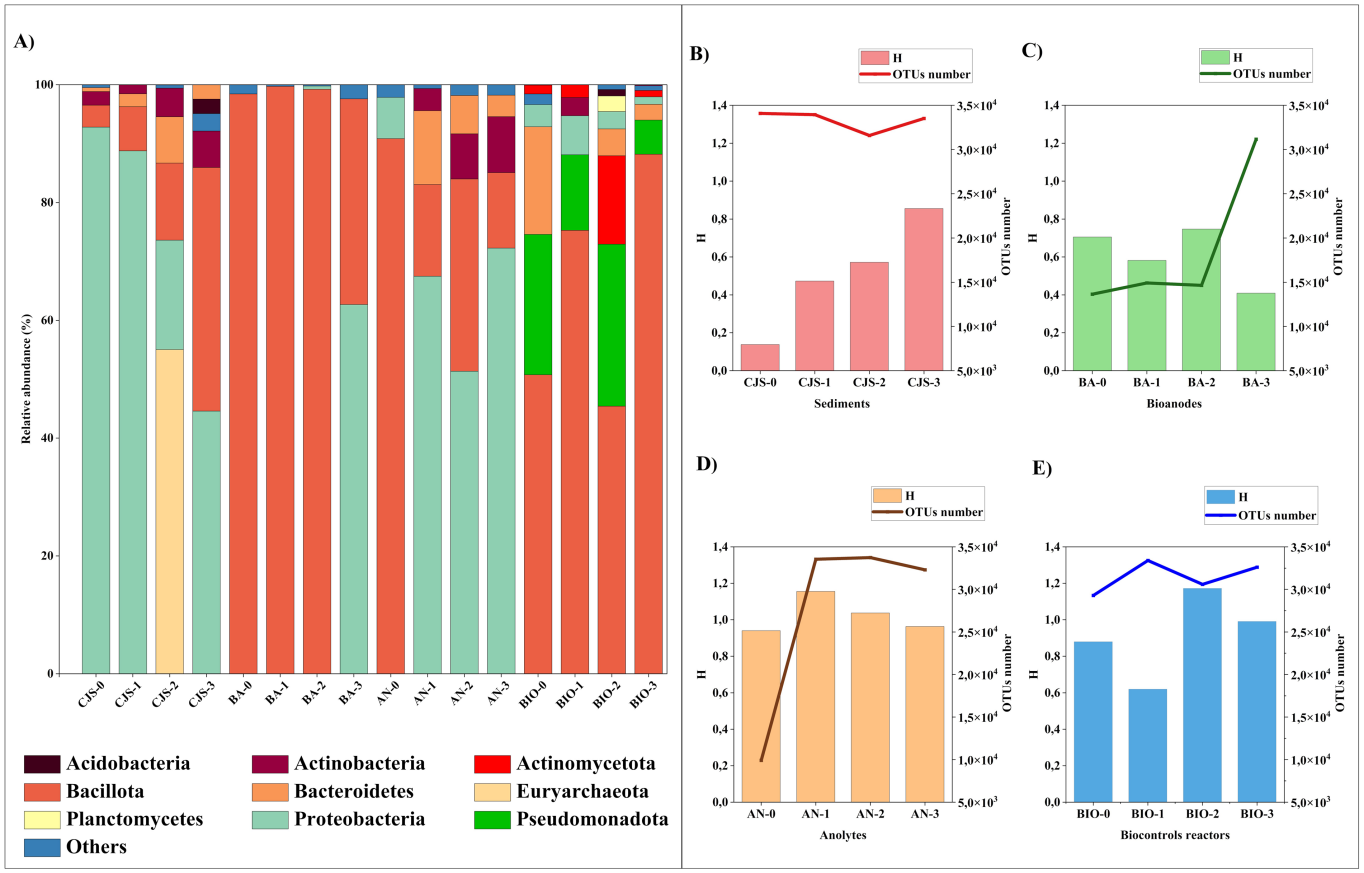
Microbial distribution at **(A)** phylum level of all samples that represented >2% in at least one sample, diversity index of microbial community Shannon (H) and the total number of OTUs for; **(B)** sediments (CJS-0, CJS-1, CJS-2, and CJS-3); **(C)** Bioanodes (BA-0, BA-1, BA-2, and BA-3); **(D)** Anolytes (AN-0, AN-1, AN-2, and AN-3) and **(E)** biocontroles (BIO-0, BIO-1, BIO-2, and BIO-3).

**Figure 7:**
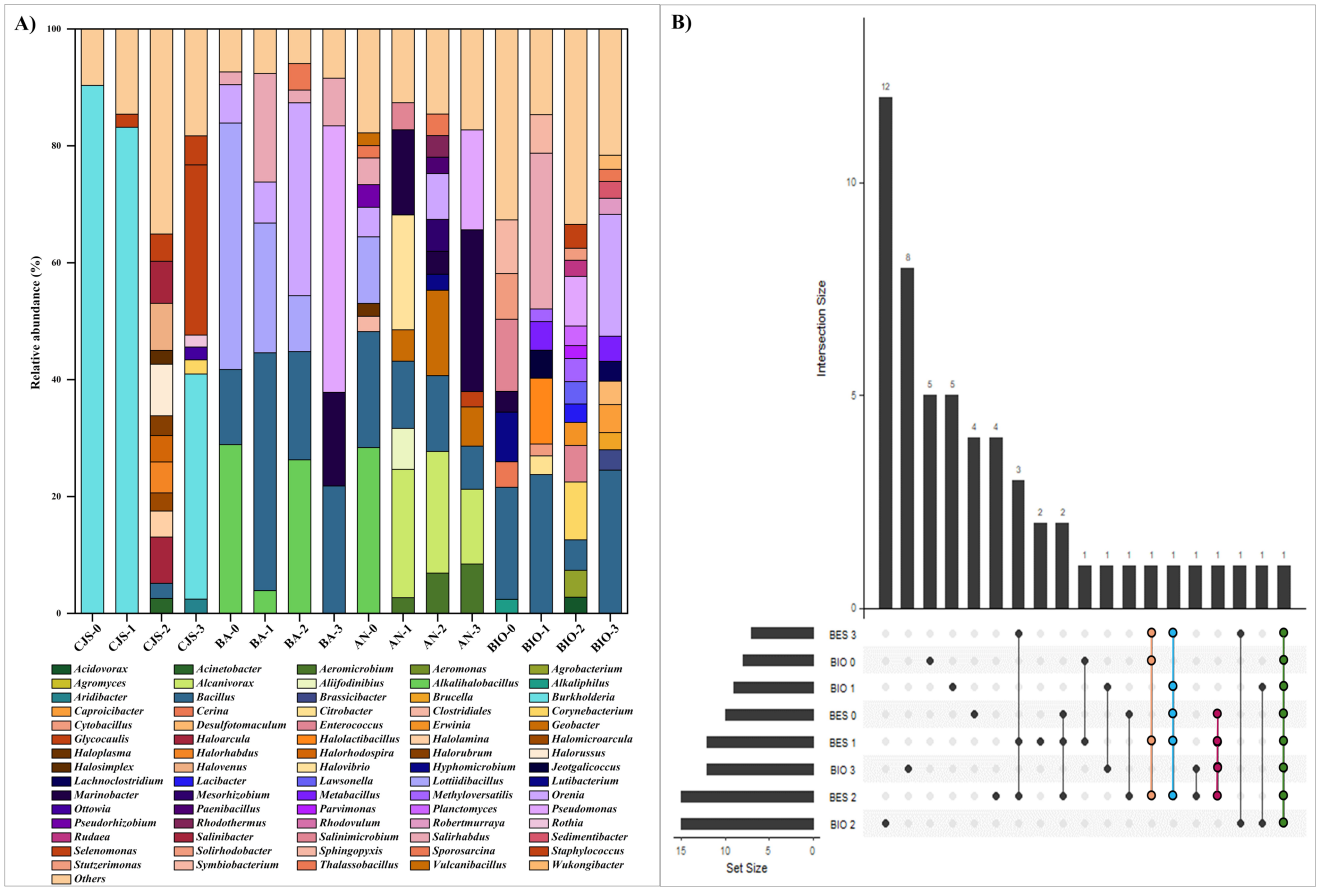
**(A)** Relative abundance of the main bacterial genus (those are more than 2%) and **(B)** UpSet plot illustrating the number of shared genera in all reactors.

The analysis of the bacterial taxonomy that shows the composition and richness of the bacterial community obtained from: sediments (CJS-0, CJS-1, CJS-2, and CJS-3), bioanodes (BA-0, BA-1, BA-2, and BA-3), anolytes (AN-0, AN-1, AN-2, and AN-3), and biocontroles reactors (BIO-0, BIO-1, BIO-2, and BIO-3) are represented in Figures 6, 7. The bacterial phyla including Acidobacteria (1.64–2.45%), Actinobacteria (1.55–9.51%), Actinomycetota (1.50–22.37%), Bacillota (also known as Firmicutes) (3.73–99.72%), Bacteroidetes (0.65–18.27%), Euryarchaeota (0.83–55.04%), Planctomycetes (3.96%), Proteobacteria (0.55–92.28%), and Pseudomonadota (5.84–41%). Only phyla with a relative abundance above 2% in at least one sample are represented (Figure 6A). Bacillota and Proteobacteria account for more than 80% of the leading nine major bacterial phyla detected in the BES samples (bioanodes and anolytes). However, Proteobacteria constitutes a minority in BIO, with a relative abundance of less than 7%, whereas Bacillota predominates with a relative abundance of more than 50%. In the BIO samples, the Pseudomonadota and Actinomycetota phyla are only found in the biosamples. However, proteobacteria are in a minority in the BIO sample, with a relative abundance of less than 7%, whereas Bacillota predominates with a relative abundance of more than 50%. In the BIO samples, the Pseudomonadota and Actinomycetota phyla are only found in the biosamples. Sequences belonging to Euryarchaeota were detected in CJS-2 (55%) but only observed in AN-2 and AN-3, with a percentage < 2%. Sequences related to Bacteroidetes observed in all CJS, were found in anolytes (AN-1, AN-2, and AN-3) and BIO (BIO-0, BIO-2, and BIO-3).

A genera-level analysis indicated a total of 381 diverse genera, with only genera having a relative abundance greater than 2%, as shown in Figure 7A. Bacterial genera included *Alkalihalobacillus* (3.89–28.89%) *Bacillus* (2.54–40.70%), *Lottiidibacillus* (9.58–42.16%), *Orenia* (5.07–32.98%), *Salirhabdus* (2.17–26.64%), *Thalassobacillus* (2.10–4.54%), *Pseudomonas* (8.50–45.60%), *Marinobacter* (3.24–24.54%), *Salinimicrobium* (3.79–12.32%), *Staphylococcus* (2.23–4.97%), and *Sporosarcina* (2.08–3.05%). Biostimulation with increasing doses of TBZ induced changes in the sediment microbial community, as shown in Figure 6B. Community richness [Shannon index (H)] increased from 0.13 for CJS-0 to 0.85 for CJS-3, while the number of OTUs remained stable. This indicates that species richness does not change, but the distribution of abundance becomes more balanced. In addition, a reduction in the dominance of the main population in CJS-0 and an increase in the equity between the present genus was observed in the relative abundance of the bacterial genus in CJS-3 (Figure 7A). The abundance of *Burkholderia* sp. decreased from 90% in CJS-0 to 7.75% in CJS-2, giving rise to enrichment populations specific to TBZ degradation, consisting of the following phyla: Bacteroidetes, Euryarchaeota, and Bacillota. The number of OTUs in bioanodes BA-0, BA-1, BA-2, and BA-3 are 0.70, 0.58, 0.74, and 0.40, respectively (Figure 6C), and in anolytes are 0.94, 1.15, 1.03, and 0.96, respectively (Figure 6D). There is constantly fluctuating despite a constant increase in the number of OTUs in BES samples (bioanodes and anolytes), which means that even with the appearance of new species, the distribution of abundances becomes unbalanced due to the dominance of some species while others remain uncommon. Figure 7A, confirming the decrease in *Alkalihalobacillus* abundance from 28% in BA-0 and AN-0 to a total absence in BA-3 and AN-0, gives the appearance of different types of bacteria that did not affect the OTUs number in those groups. The response of BIO to biostimulation (Figure 6E) is similar to the BES response. However, when the Shannon index decreases, the number of OTUs increases. It means that, while there are more genera, their relative abundance is highly unequal, with a few taxa dominating the rest. BES and BIO reactors were compared in the form of an UpSet plot to illustrate the shared genera in all reactors (Figure 7B). The core analysis revealed that the only core genera in all reactors were *Bacillus* (green dots). Out of these, *Marinobacter* and *Salirhabdus* were identified in 4 and *Orenia* in three different reactors. 4, 2, 4, 5, 5, 12, and 8 genera were presented exclusively in BES-0, BES-1, BES-2, BIO-0, BIO-1, BIO-2, and BIO-3 reactors, respectively. Moreover, BES-3 shares at least one genus with the other BES and BIO reactors which were composed of *Aeromicrobium*, *Alcanivorax*, *Bacillus*, *Geobacter*, *Glycocaulis*, *Marinobacter*, *Pseudomonas*, and *Salirhabdus*.

## 4 Discussion

In this study, we first examined the characteristics of CJS, recognizing the importance of the inoculum source for BES performance. CJS conductivity was 74.1 mS cm^−1^, which is higher than the average seawater conductivity of 50 mS cm^−1^ (Ashokan et al., 2023) and notably larger than that of FPWW (2.9 ± 0.3 mS cm^−1^) (Rodrigues et al., 2022). The pH of CJS was 7.8, and in the reactors, the final pH stabilized at 7.0 ± 0.1 closely resembles that of real FPWW, which can reduce the adaptation period for the inoculum when transferred to actual FPWW, promoting optimal microbial function without the need for additional condition adjustments. Moreover, the chemical analysis of the sediments, focusing on the nutrient profile, including organic carbon, phosphates, and minerals, demonstrates the richness of CJS. This may enhance microbial growth, leading to improved performance in BES.

### 4.1 Impact of TBZ concentration and glucose on current production and TBZ removal efficiency

As far as the measurement of current density using the same sediments sampling site (Chott El Dejrid), bioanodes generated with BES-0 had less success than those obtained in our prior research (Saidi et al., 2022). Indeed, the highest current densities recorded for bioanodes in this study were below 1 mA m^−2^ (BES-0) when a concentration of 150 ppm of TBZ was present, along with 30 ppm of glucose as co-substrate. In contrast, using 50 ppm of TBZ without adding the co-substrate, bioanodes in our previous study generated 3.66 mA m^−2^. However, after 7 days of polarization, the TBZ removal rate in BES-0 was less than 25%, corresponding to 37.50 ppm (Figure 6B). This is close to the concentration of TBZ degraded in 7 days in our earlier study, where there was a decrease of over 80% in TBZ concentration corresponding to 40 ppm. These discrepancies observed between these two experiments could be explained by the nature of carbon sources. Glucose is quickly consumed by microorganisms, and its presence may impact the microbial community dynamics. Due to its fermentable nature, glucose can be converted into volatile fatty acids, which are then utilized by microbes (Guo et al., 2022). This fermentation process leads to changes in bacterial metabolism, prompting glucose consumption and utilizing electrons produced to degrade TBZ while downregulating other enzymes responsible for directly consuming TBZ. Furthermore, Guo et al. (2022), identified that *Acetobacterium* was enriched on the electrode surface only in the presence of glucose. Although this bacterium was not involved in azo dye reduction, it is recognized as an acetogenic microbe capable of producing acetate through glucose oxidation. This highlights the differing impacts of using two distinct substrates as electron donors. While we achieved our primary goal of TBZ degradation, we could not increase current densities by adding a co-substrate. Nevertheless, the noticeable decrease in the current production can be related to the elevated concentrations of TBZ. It could inhibit the microbial activity to electron transfer, resulting in a lower current density. Shahi et al. (2020) observed a similar phenomenon while degrading a recalcitrant molecule, the Reactive Orange 16 Dye. They reported that at high dye concentrations, current production was inhibited, and COD removal decreased in the microbial fuel cell (MFC) once the dye concentration reached 500 ppm. More recently, a study carried out by Saadaoui et al. (2023b) showed that the BES performance was reduced as the dye concentration was increased for two different dyes used. These outcomes align with our results, where we successfully degraded TBZ at an initial concentration of 50 ppm (in our previous study). In the current study, certain microorganisms could be selectively inhibited while increasing the TBZ concentration in the reactor (150 ppm). This, in turn, can negatively impact BES performance and reduce the overall effectiveness of microbial activity leading to a decrease in current production.

### 4.2 Biostimulation effects

A significant change in current production occurs after the first 15 days of biostimulation in the presence of 10 mg of TBZ per kg of sediment. The current maximum output declined to 0.4 mA m^−2^, raising concerns about the impact of biostimulation on cell performance. Biostimulation may alter the microbial community’s composition and diversity, potentially favoring the proliferation of competing microorganisms that lack the metabolic capacity to contribute effectively to the current generation. This was demonstrated by an increase in the relative abundance of *Aeromicrobium* genera and the appearance of *Glycocaulis* genera in AN-3 (Figure 7A). Both of these are aerobic microbes known for their ability to degrade aromatic compounds, including mycotoxins (Hu et al., 2023) and hydrocarbons (Chen et al., 2024). However, they have not been identified as electroactive bacteria. After the initial biostimulation with a higher TBZ dose and using CJS-2 as inoculum, a notable drop in current production was noticed, followed by increased current densities (Figure 2D). The electrocatalytic improvement from BES-0 to BES-2 corresponded to an amplification factor of three, reaching a maximum current density of 3.22 ± 0.58 mA·m^−2^. Considering the boost in intensity observed using inoculum from the second biostimulation sediment (CJS-2), we can note that microorganisms require time to acclimate to the unexpected stimuli provided while undergoing physiological changes to adapt to the new environment. This biostimulation period enables microorganisms to increase their enzymatic activity for TBZ degradation by genetic recombination and natural selection to favor species that contribute to forming a microbial community capable of breaking down TBZ (Wani et al., 2022). After the third stage of biostimulation, BES-3 produced three peaks of current with different intensities, achieving a peak with a maximum current density of 2.00 ± 0.17 mA m^−2^, which was twice the amplification seen in BES-0. Even though the BES-3 output with CJS-3 as the inoculum was higher than the non-biostimulated sediment CJ-0, it remained lower than the CJS-2 biostimulation. This might be attributed to the reduced ability of microorganisms to produce current due to the changes in the microbial community structure, including shifts in the proportions and diversity of existing microorganisms after the third biostimulation cycle, potentially favoring species that cannot produce current. Alternatively, some modifications in the metabolic activities of the microbial consortium to resist the fungicide could impact their ability to generate current.

### 4.3 Metabolic shift and its influence on electron transfer and TBZ degradation

Electroactive bacteria can adapt their metabolic pathways based on environmental conditions. Facing the high concentrations of TBZ, these microorganisms could shift their metabolism from electrogenic processes to fermentative metabolism. This shift leads to a decrease in electron transfer activity and an increase in energy consumption, as the bacteria boost their metabolic activity for TBZ degradation and to combat the excessive and toxic concentrations of TBZ. Consequently, the balance of NADH/NAD+ changes throughout this metabolic shift, which can significantly affect the bacteria’s ability to use organic compounds as the final electron acceptors (Moscoviz et al., 2016). In addition, an increase in the redox couple NADH/NAD+ helps to promote fermentation. Although this alteration may reduce the overall efficiency of BES for energy generation, allows bacteria to resist excessive TBZ concentrations. Therefore, it is essential to clarify these metabolic dynamics to optimize BES and enhance the productivity of the microbial communities dedicated to TBZ degradation. Despite the variation in current production from different BESs using biostimulated CJS, the improvement in TBZ degradation is quite remarkable, 39.68, 84.03, and 88.33% for BES-1, BES-2, and BES-3, respectively, after 7 days of polarization. This increased rate of TBZ degradation could be explained by the enrichment of BES with multiple bacteria, including fermentative bacteria, which do not adhere to the anodes and consume TBZ in the anolyte. Also, it may be possible that these types of bacteria utilize the electrons produced by electrogenic bacteria from glucose metabolism to facilitate the fungicide degradation. In addition, a total degradation of glucose was observed within 4 days in all reactors, which could theoretically give 308°C. However, the accumulated charge in all BESs ranged from 0.88 to 5.69\u00B0C, which is lower than the expected value based on glucose molecules. Such results could be due to the lack of effective transfer of electrons in BES or the deviation of produced electrons to other biochemical processes, that electron-receiving bacteria could use for the reduction of TBZ. Microorganisms may use glucose for alternative metabolic pathways, such as fermentation or anaerobic respiration, reducing the number of electrons available for electricity generation at the BES anode. This interaction among the microbial community, specifically between electrogenic and electrotrophic bacteria, allows synergistic interplay via extracellular electron transfer, to enhance the degradation of pollutants. Song et al. (2019) used BES inoculated with industrial sludge to degrade p-chloronitrobenzene and enhanced the degradation process through a bioaugmentation approach. They added *Pseudomonas* sp. as a biocatalyst, which allowed them to improve pollutant removal to 100%. This demonstrates that the degradation efficiency of pollutants can be enhanced via by introducing specific microorganisms. Hence, the involvement of exoelectrogenic bacteria is crucial, as they act as electron donors, facilitating the biodegradation process. This demonstrates that introducing specific microorganisms can enhance the degradation efficiency of pollutants. It is crucial to note that CJSs have demonstrated their potential performance in BES to design efficient bioanodes from various substrates (Askri et al., 2019, 2020; Saadaoui et al., 2023a, 2023b). While current production depended is on the substrate nature, effective removal of the pollutant was achieved (Saidi et al., 2022). These outcomes point out that CJS may be a valuable asset for developing effective, ecofriendly pollutant removal methods using BES.

Biofilms formed on the WE electrodes’ surface are characterized by an amorphous structure that corresponding to an enveloping matrix consisting mostly of extracellular polymeric substances and proteins released by microorganisms to promote their adhesion to the material’s surfaces. Such structures are associated with the mechanisms of electron transfer in energy-producing processes in BES (Agudelo-Escobar et al., 2022). However, a sparse biofilm structure was observed where the microbial adhesion occurred only between the fibers of carbon felt, suggesting a few numbers of bacteria adhered on the anode. This weak attachment could contribute to a lower electron transfer to the working electrode surface (Zhang et al., 2019), leading to the obtained low current production in chronoamperometry’s results discussed above. Furthermore, the small current amount generated with electroactive biofilms could be related to the elevated resistance to charge transfer occurring at the anode, which can result from limited biofilm development on the bioanode, as suggested by Kabutey et al. (2019). When the biofilm is not well developed, fewer electroactive microorganisms are in direct contact with the anode surface, reducing electron transfer efficiency. This increased resistance can impede the flow of electrons, decreasing the overall current generated. A fully developed biofilm is key in facilitating an efficient electron transfer between bacteria and the anode surface. Limited biofilm formation, as shown in our results, could reduce the number of electroactive bacteria in contact with the anode, directly impacting electron transfer efficiency. Overall, the low currents obtained in our study, which were less than 4 mA m^−2^ consistent with those reported in a previous study on the removal of persistent chemicals (Wu et al., 2017).

GC-FID results demonstrate that within the first 5 days of polarization at 0.1 V vs. SCE, the TBZ removal rate increased in BES-0 by a factor of 5 compared to BIO-0, with removal rates of 16 and 3%, respectively. This aligns with our prior finding (Saidi et al., 2022), in which a weak electrostimulation of CJS in BES increased TBZ degradation by approximately 40% over biological degradation alone. This demonstrates the reproducibility of the results and strengthens the idea of using electrostimulation to enhance TBZ degradation in BES compared to conventional bioprocesses significantly. However, over time, this initial gap diminished, and at the end of the experiment (day 35), BES-0 achieved an elimination rate of 94%, compared with 84% for BIO-0, giving an increase factor of approximately 1.12. Despite this trend, BES maintains a slight edge over biological degradation, particularly at concentrations above 15 ppm, as observed in the final phase of the experiment. Several studies have demonstrated the effectiveness of electrostimulation in improving the breakdown of recalcitrant substances. Wang et al. (2022) developed a microbial electrolysis cell coupled with biotrickling filters to improve the removal efficiency and elimination capacity of gaseous m-dichlorobenzene (m-DCB). With weak electrical stimulation, researchers achieved 1.48- and 1.65 times higher m-DCB removal than the biological process alone. In addition, Abudureheman et al. (2023) demonstrated the positive effect of intermittently applied voltage (0.9 V) on phenol fluoroquinolones (ofloxacin, norfloxacin, ciprofloxacin, and enrofloxacin) degradation rates by selectively stimulating electroactive bacteria related to biodegradation, contributing to promoting the degradation of target pollutants. Compared to the biological degradation process, BES is more efficient in removing TBZ, potentially due to the enhanced metabolic activities of enriched microorganisms. Within the first biostimulation of CJS with 10 mg kg^−1^ of TBZ, the TBZ removal was observed in BIO-1 compared to BIO-0 after 5 days from using the stimulated CJS-1 as inocula. Hence, 5 days after the beginning of the experiment, biostimulation strategy appears to be also effective in improving TBZ removal. However, no significant difference was observed between BES-0, BES-1, and BIO-1, which were 15.90, 16.12, and 12.98%, respectively. This could be due to the alteration of the indigenous microbial community leading to changes in the population of electroactive bacteria by decreasing their number and, therefore, to less effective electrostimulation in BES-1 caused by the change in TBZ degradation pathways as explained in bacteria community analysis. This is demonstrated by the Taxonomic results, where we can see the increase in the population of *Salirhabdus* sp. from 2.17 in BA-0 to 18.57% in BA-1, the decrease in *Alkalihalobacillus* sp. from 28.89 in BA-0 to 3.89% in BA-1 and the appearance of various bacteria in AN-1 and BIO-1 known for their potential metabolism in the bioremediation of various toxic compounds such as crude oil and tannery waste (Lukhele et al., 2022; Liu et al., 2023). On day 7, a noticeable improvement in elimination rates was noted between BES-1 and BIO-1, with BES-1 eliminating at 39.68% compared to BIO-1’s removal rate of 26.75% giving an improvement factor of approximately 1.5.

Furthermore, after 35 days of biostimulation, the elimination rate of BES-1 only exceeds that of BIO-1 by a factor of 1.03. This could be explained by the decrease in TBZ concentrations, which limits its availability in the reactors. Approximately 46.80% of TBZ was degraded within just 5 days in BES-2, with 41.11% in BIO-2 after the second biostimulation using CJS-2. This result indicates a slight enhancement in TBZ degradation compared to previous conditions by effectively stimulating microbial activity leading to accelerated TBZ removal. At the end of the 35-day experiment, the BES-2 and the BIO-2 showed exceptional effectiveness in eliminating TBZ from the synthetic FFWW. BES showed a slightly greater removal rate of 99.77% than BIO’s 98.15%. BES-3 demonstrated a notable removal rate of 38% which is significantly higher than that of BES-0 after 5 days, while BIO-3 showed a slightly lower rate of 29%. This proves the efficiency of sediment biostimulation in enhancing TBZ degradation, even in biological processes. After the second biostimulation, we observed a real improvement in bioremediation. Furthermore, the degradation rate varied significantly between the non-stimulated and stimulated sediments (Figure 4). BES-0 offers a quicker TBZ degradation rate than the biological reactor (BIO-0), having half-lives T_(1/2)_ approximately 19 and 27 days, respectively (Table 2). Nevertheless, coupled biostimulation and electrostimulation in BES-3 contribute to a considerable rise in the degradation rate with a half-life of 5.5 days, illuminating a substantial boost of TBZ decay. Our results confirm that combined biostimulation of CJS to the electrochemical stimulation in BES can helpfully increase the activity of microorganism degradation. Similarly, Bhardwaj et al. (2020) demonstrated the effectiveness of biostimulation with molasses in enhancing the degradation of atrazine (herbicide), achieving 90% atrazine removal in 13 days, a much faster rate than natural attenuation which requires 42 days to reach the same level of degradation. Furthermore, tebuconazole (fungicide) degradation was more intense using organic biostimulating substance (bird droppings) boosting enzyme activities of dehydrogenases, catalases, alkaline phosphatase, acid phosphatase, and arylsulfatase (Baćmaga et al., 2019). Our research showed that biostimulation of hypersaline sediments by adding TBZ had a notable impact on the overall degradation activity of TBZ, allowing for the rapid and efficient removal of this fungicide. When using the biostimulation approach, BESs were able to remove TBZ within 25 days completely. Further research is essential to gain a deeper understanding of the mechanisms involved in TBZ degradation to enhance treatment methods for FPWW contaminated with TBZ. FTIR results indicate the disappearance of peaks from 1,500 to 400 cm^−1^, which are the fingerprints of the TBZ molecule. This clearly shows that TBZ was broken, generating transformation products in the BES inoculated with either the biostimulated or the non-biostimulated CJS, supporting the results obtained from UV–visible and GC analyses.

### 4.4 Electrogenic properties of Bacilli class members

Taxonomic analysis revealed that Bacillota (also known as Firmicutes) is the highest represented phylum in biofilms colonizing the bioanodes BA-0, BA-1, and BA-2 including *Alkalihalobacillus* (3.89–28.89%), *Bacillus* (12.83–40.70%), *Lottiidibacillus* (9.58–42.16%), *Orenia* (6.57–32.98%), and *Salihrhabdus* (2.17–18.57%) genera. These genera are the members of the Bacilli class which has been recognized for their capacity to break or transform a wide range of chemicals, such as dyes, drugs, pesticides, heavy metals, explosives, aromatic acids, and various other toxic substances (Arora, 2020). In addition, in our previous study (Saidi et al., 2022), *Bacillus* genera were also identified, and it had been correlated with the TBZ degrading consortium. Serval enzymes responsive to the stress caused by pesticide exposure, such as esterase aldehyde dehydrogenase and laccase have been previously discovered in this genus. These enzymes can effectively degrade various pesticides, including organophosphate, carbamate, and organochlorine compounds (Liaqat et al., 2023). Despite being considered potential bioremediation agents, members of the Bacilli class are identified as weak electroactive bacteria (Su et al., 2020). They are associated with electrofermentation processes, that can be utilized in the synthesis of fine chemicals (Schievano et al., 2016). Several studies have reported that Bacilli cells use cytochromes and NAD+/NADH pathways for extracellular electron transfer, with flavin involvement as a redox mediator (Higashitsuji et al., 2007; Chen et al., 2019). This mechanism could improve their metabolism to withstand the stress caused by toxic compounds such as TBZ and increase their ability to detoxify the polluted environment. This kind of biofilms formed by members of the Bacilli class have been found with poor electrogenic activity in various environments (Chabert et al., 2015). Hence, the loss of current densities occurred in BES-0, BES-1, and BES-2, which is observed by the losses of the accumulated charge (Qmax, Table 3) in those reactors compared to the theoretical values given by the consumption of glucose, could be attributed to Bacilli class.

### 4.5 Synergistic role of electroactive and non-electroactive bacteria in TBZ degradation

The first evidence of fungicide biodegradation by *Orenia* of the class Clostridia was reported in this study. *Orenia* sp. was discovered in deep subsurface environments and is notable for its ability to reduce iron minerals (Dong et al., 2016) and fermenting Carbohydrates (Mouné et al., 2000) while no report on the electrogenic potential of this genus is provided in the literature. Saadaoui et al. (2023a) previously cited the dominance of this genus and its presence in all samples, proving its role in the degradation of azo dyes. However, a different scenario showed up in BA-3, where the genera *Alkalihalobacillus*, *Lottiidibacillus*, and *Orenia* were absent and replaced by Proteobacteria phylum (62.68%). This shift has been associated with the enrichment of *Marinobacte*r and *Pseudomonas* and several other genera in the bioanode. *Marinobacter* sp. (16%) is involved in many biodegrading processes of recalcitrant aromatic compounds. They have been demonstrated to degrade several kinds of hydrocarbons (Rushimisha et al., 2023) and dyes (Saadaoui et al., 2023b), presenting them as suitable bacteria for bioremediation projects. In addition to that, *Pseudomonas* (45.6%) was the dominant genus in BA-3. These kinds of bacteria are widely known for their exoelectrogenic ability, which can transfer electrons produced by the oxidation of organic compounds to the anode (Kaushik and Jadhav, 2017; Song et al., 2019). Moreover, these bacteria proved their potential to degrade various aromatic compounds, such as dyes (Kishor et al., 2021; Saadaoui et al., 2023a), hydrocarbons (Sawadogo et al., 2024), and pesticides (Perruchon et al., 2015; Song et al., 2019; Liu et al., 2022). Papadopoulou et al. (2018) reported the abundance of *Pseudomonas* sp. in soil samples contaminated by high levels of TBZ and their resilience to this fungicide, giving them a strong ecological advantage to survive and actively multiply in an environment with limited niche competition. According to this scenario, *Marinobacter* and *Pseudomonas* should provide remarkably efficient electroactive species. However, they cannot form a structured biofilm capable of transferring electrons to the anode surface, as observed in the CLSM 3D image. Thus, this phenomenon could be explained by the consumption of the exopolymer matrix by various anolyte bacteria to activate their metabolisms to degrade or resist TBZ concentrations—particularly, as reported by Feng et al. (2022).

### 4.6 Biochemical mechanisms of *Pseudomonas* in aromatic compound degradation

*Pseudomonas* species can degrade benzene rings using dioxygenase enzymes under aerobic conditions. These enzymes catalyze the cleavage of the aromatic ring, producing *cis*-dihydrodiol as an intermediate. Then, this intermediate is converted to NAD+ by dehydrogenase as part of the metabolic process. Meanwhile, catechol, another essential intermediate, is further metabolized. Finally, hydrolase enzymes cleave the catechol ring at the ortho position, forming *cis*, *cis*-muconic acid. This series of biochemical reactions shows the ability of *Pseudomonas* sp. to efficiently metabolize aromatic compounds, underlining their potential for bioremediation applications.

### 4.7 Competition for anode surface

Whereas *Geobacter* is known for its ability to perform extracellular electron transfer by adhering to electrode surfaces in microbial electrochemical systems (Hu et al., 2021), this genus was observed only in anolytes after biostimulation; AN-1, AN-2, and AN-3. Its absence on bioanodes could be due to the competition with other microbes; most likely electrogenic bacteria can compete for the limited space available on the anode surface (Abadikhah et al., 2022). The bacterial genera *Alkalihalobacillus*, *Bacillus*, *Lottiidibacillus*, *Salihaerhabdus Pseudomonas*, *Orenia*, and *Marinobacter* represent diverse groups of bacteria that share the ability to be aerobic or facultative anaerobes which is not the ideal environment for the anaerobes *Geobacter*. Thus far, it has been reported that Geobacter is capable of transferring electrons obtained from substrate oxidation to carry out the extracellular reduction of nitroaromatic compounds, ultimately aiding in the conversion of nitro groups into amino aromatics with decreased toxicity (Feng et al., 2022). This demonstrates the population’s capability to break down TBZ while using glucose. The abundance of the Bacteroidetes phylum increased in the sediments after the two first stages of biostimulation, but it decreased after the last phase. This trend suggests that TBZ initially stimulated this population when used at doses ranging from 10 to 100 mg kg^−1^. However, as the TBZ concentration increased in CJS-3, it inhibited bacteria proliferation and may become toxic to the Bacteroidetes population. This effect was further demonstrated in reactors where the abundance of Bacteroidetes phylum decreased in anolytes (from 12.51 to 3.62%) and in the BIO (from 18.27 to 2.69%), when using CJS with increasing doses of TBZ, in combination with the 150 ppm of TBZ presents in reactors. The third biostimulation cycle in the BIO-3 shifts in microbial communities led to a marked change in microbial communities, resulting in the dominance of Bacillota bacteria (increasing from 45.09 to 81.05%) and the inhibition of Pseudomonadota (from 23.87 to 5.84%) and Proteobacteria (from 3.78 to 1.28%).

### 4.8 Future insights

A significant shift in bacterial community structure due to biostimulation was observed at the genus level, with notable changes in bacterial composition occurring after each cycle. These significant shifts were also observed in BIO as demonstrated by the UpSet plot (Figure 7B). While these changes enhanced TBZ degradation, a slight gap remained compared to BES, highlighting the value of electrostimulation in selecting bacteria profiles with maximum microbial diversity. This diversity enhances the resilience and adaptability of the community to environmental changes and stressors, such as the presence of contaminants like TBZ. Therefore, it is essential to investigate the causal connection between biofilm electroactivity, the degradation of aromatic recalcitrant compounds, and the generation of metabolites. Additionally, exploring how biofilm-based bioprocesses might be improved through the application of specific electrical potentials could provide valuable insights (Eghtesadi et al., 2022).

## 5 Conclusion

This study demonstrates the effectiveness of weak electrostimulation with BES in degrading 94% of the aromatic recalcitrant compound TBZ using CJS as inocula, after 35 days. Interestingly, an enhancement of the BES treatment efficiency was observed when coupled to biostimulated CJS as inocula. Significant shifts in microbial dynamics composition were established, with an enrichment of the Proteobacteria phylum. This change in bacterial community structure was associated with an increase in TBZ removal and degradation rate exceeding, 99% within 25 days, along with a rise in current densities. Despite challenges such as limited electron transfer at the anode, these findings provide valuable insights for optimizing bioremediation strategies to achieve more efficient pollutant removal in FPWW.

Future in-depth investigations should explore the mechanisms behind microbial electrochemical TBZ degradation in BES, with particular attention to microbial community interactions and their roles in enhancing pollutant removal. Additionally, integrating advanced materials and innovative electrochemical approaches could further improve the performance of BES in the current generation. The results of this study open new avenues for developing sustainable, efficient BES with potential applications in wastewater treatment, environmental remediation, and renewable energy generation.

## Data Availability

The original contributions presented in the study are publicly available. This data can be found in the https://www.ncbi.nlm.nih.gov/bioproject/PRJNA1198214, with the following accession numbers: SAMN45833250 -SAMN45833265.

## Footnotes

^1^ www.mrdnalab.com

^2^ www.ncbi.nlm.nih.gov
